# Surface water circulation develops seasonally changing patterns of floating litter accumulation in the Mediterranean Sea. A modelling approach

**DOI:** 10.1016/j.marpolbul.2019.110619

**Published:** 2019-12

**Authors:** D. Macias, A. Cózar, E. Garcia-Gorriz, D. González-Fernández, A. Stips

**Affiliations:** a European Commission, Joint Research Centre, Via E. Fermi 2749, 21027, Ispra, Varese, Italy; b Departamento de Biología, Campus de Excelencia Internacional del Mar (CEIMAR), Instituto Universitario de Investigaciones Marinas (INMAR), Universidad de Cádiz, E-11510, Puerto Real, Spain

**Keywords:** Numerical modelling, Surface circulation, Floating litter, Mediterranean sea

## Abstract

Marine litter and, particularly, plastics are a growing concern at global scale. The Mediterranean Sea is among the zones in the world with the highest concentration of floating plastic debris. However, our knowledge remains limited on the spatial distribution of litter across this basin. Here, a set of different numerical model simulations were conducted to examine the dynamic conditions of the surface layer of the Mediterranean and how this drives the circulation and accumulation of floating litter. Seasonal dynamics of surface water circulation led to contrasting distribution patterns of floating litter along the year. Multiple hot spots of litter zones appeared across the basin in summer, while litter disperses and moves towards the Eastern Mediterranean and nearshore waters in winter. Taking into account such seasonal variability in the spatial patterns of litter in the Mediterranean seems to be key in the design of further sampling surveys and management strategies.

## Highlights

• Surface current dynamics shows marked seasonality in the Mediterranean.
• Accumulation and distribution of floating particles follow currents patterns.
• Floating litter accumulates in diverse areas during different seasons.
• Lagrangian model helps to identify beaches impacted by floating litter.

## 1 Introduction

Plastic pollution of marine ecosystems has become a world-wide threat. Plastic litter hardly biodegrades in nature, weathering only breaking down large plastic objects into abundant small pieces commonly called microplastics (Andrady, 2011). This persistent form of plastic pollution is spreading out by water currents over every ocean and sea on Earth, from temperate to polar regions (Law et al., 2010; Cozar et al., 2004; 2017).

Microplastics are about the size of plankton, therefore becoming available to be accidentally ingested by a wide range of marine animals (Lusher et al., 2013; Hall et al., 2015; Van Cauwenberger et al., 2015). They can contain contaminants added during plastic manufacture or acquired from the surrounding environment (Koelmans et al., 2011), which raises concerns about the potential transfer of plastic-associated contaminants throughout marine food webs (Mato et al., 2001) and even humans (Koch and Calafat, 2009; Talsness et al., 2009).

The Mediterranean Sea seems to be particularly vulnerable to plastic pollution. The semi-enclosed nature, high coastal population (Jambeck et al., 2015), intense touristic and maritime activities and anti-estuarine general circulation (Macías et al., 2016) turns the Mediterranean into an accumulation basin from where floating litter can hardly escape (Cozar et al., 2015). Further, the Mediterranean Sea represents less than 1% of the total ocean area but accumulates huge amounts of marine plastic (Cozar et al., 2015; van Sebille et al., 2015) and hosts a disproportionately large biodiversity (between 4% and 18% of all marine species; Bianchi and Morri, 2000).

Using a global dataset of floating plastic debris to calibrate three different ocean circulation models, van Sebille et al. (2015) established the range of plastic abundance in the Mediterranean surface waters between 3 and 28 trillions particles, representing 21%–55% of all floating particles in the global ocean (vanSebille et al., 2015). In mass, the Mediterranean surface plastic load was estimated to range from 5 to 30 thousand metric tons, equivalent to 5% and 13% of the global surface plastic load, respectively. The wide differences between the global fraction of Mediterranean plastic in abundance (21%–50%) and weight (5%–13%) implies that plastic debris should be considerably small and light in the Mediterranean, although this feature does not fit well with field observations (Cozar et al., 2015). Furthermore, Mediterranean surface plastic loads directly estimated from field measurements converge on range within 1–3 thousand metric tons (Cozar et al., 2015; Ruiz-Orejon et al., 2016; Suaria et al., 2016), considerably lower than the range estimated from models calibrations. This mismatch is, however, not surprising as models do rely on poorly constrained initial and boundary conditions for litter inputs and most do not incorporate some key loss processes (sinking, beaching, breaking down) that remove plastic debris from the surface ocean. On the other hand, substantial uncertainty is still attached to field measurements, in large part due the sparse nature of the ship surveys and the apparent spatio-temporal variability of the floating litter. Numerical models require better field information, while a better understanding of the distribution and circulation patterns acquired through modelling may benefit the sampling strategies of the ship surveys. Strengthen this synergy is an avenue to improve our knowledge of the problem of the marine litter.

Numerical models have identified contrasting patterns of surface plastic distribution for the Mediterranean basin (*e.g.,* Lebreton et al., 2012; Maximenko et al., 2012; Poulain et al., 2012; Mansui et al., 2015; van Sebille et al., 2015; Zambianchi et al., 2017; Liubartseva et al., 2018). In certain cases, regions within the North-Western Mediterranean Sea have been identified as potential litter accumulation zones (Maximenko et al., 2012; Liubartseva et al., 2018). Some simulations, have pointed to the south and central Ionian Sea as accumulation areas (Lebreton et al., 2012; Liubartseva et al., 2018), while the Eastern Mediterranean is estimated to accumulate substantial amounts of floating litter in other studies (Lebreton et al., 2012; van Sebille et al., 2015). However, to date, field surveys have failed to confirm the presence of permanent accumulation areas in the Mediterranean Sea. High mesoscale activity and wide yearly variability seem to explain the inconsistencies between model outputs and field data (Cozar et al., 2015; Suaria et al., 2016; Ruiz-Orejon et al., 2016). Also, most of the models referenced here have a spatial resolution that's eddy-permitting but not eddy-resolving, while in the Mediterranean Sea, mesoscale variability is known to be extremely large (Millot and Taupier-Letage, 2005). The high intensity mesoscale field makes permanent or climatological features prone to be disrupted, so, in order to correctly understand and predict circulation and accumulation patterns of floating litter, adequate resolution models are needed.

Understanding how floating litter distributes across the Mediterranean Sea is of paramount importance to assess the magnitude and potential impacts of the plastic pollution on this unique ecosystem. In order to improve our competences to model transport of plastic debris, here we aim to identify gaps in the current modelling approaches by analyzing the variability at the seasonal scale and the connections between water current dynamics and accumulation patterns. For this purpose, we use the Marine Modelling Framework (MMF, Stips et al., 2015) developed at the Joint Research Centre (JRC) of the European Commission forced by a long (2000–2017) and high frequency (6-hourly) time-series of realistic atmospheric conditions. We expect that this analysis will provide a useful background to guide future modelling developments and observational works.

## 2 Material and methods

### 2.1 Models description

To simulate the hydrodynamic conditions in the Mediterranean Sea, we have used the General Estuarine Transport Model (GETM, Burchard and Bolding, 2002) within the Marine Modelling Framework (MMF, Stips et al., 2015). The MMF is the Regional Earth System Model developed at the EU Commission Joint Research Centre (Ispra, Italy). For this study, we have simulated 18 years (2000–2017) of hydrodynamic conditions in the basin.

**Fig. 1:**
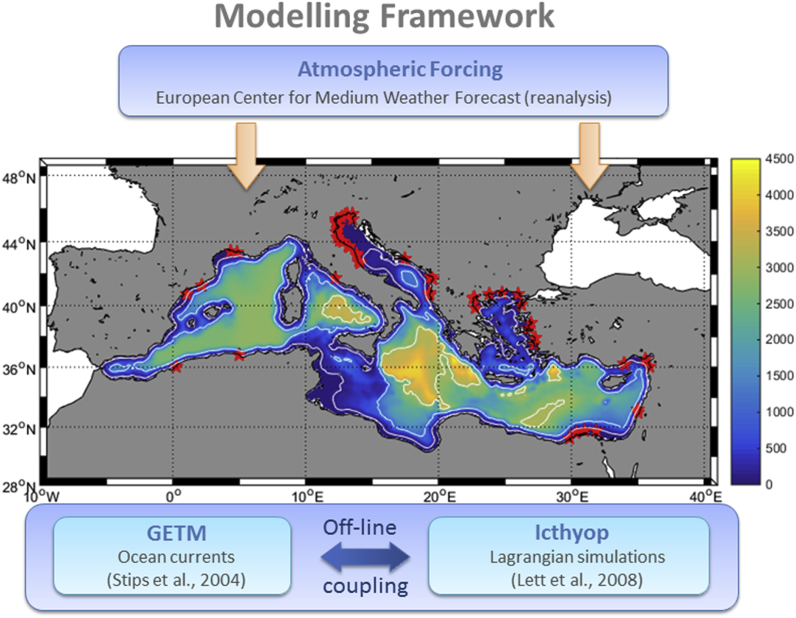
Marine modelling framework scheme in the present implementation. Red stars mark the position of the rivers included. (For interpretation of the references to colour in this figure legend, the reader is referred to the Web version of this article.)

This particular GETM implementation (Fig. 1) has a horizontal resolution of 5′ x 5' (∼9 × 9km) and includes 25 vertical sigma-layers. Model bathymetry was built using ETOPO1 (http://www.ngdc.noaa.gov/mgg/global/) database while initial and boundary thermohaline conditions were created by using the Mediterranean Data Archeology and Rescue-MEDAR/MEDATLAS database (http://www.ifremer.fr/medar/). Data from the European Center for Medium-Range Weather Forecast (ECMWF-ERAin) with a frequency of 6 h were used to force GETM at the surface while the largest 53 rivers were simulated to flow into the Mediterranean Sea basin. River discharges were taken from the Global River Data Centre (GRDC, Germany) database.

Lagrangian simulations were performed using the free modelling tool Ichthyop v.3.3 (available at http://www.ichthyop.org) coupled off-line with the ocean model described above. Six-hours ocean simulations were used to perform the off-line coupling with Ichthyop. Each litter particle was simulated as a virtual inert drifter floating at the sea surface being advected by the surface currents (computed by the GETM model). For a full description of the Ichthyop tool and its capabilities the reader is referred to Leet et al. (2008) and to the documentation provided at http://www.ichthyop.org/. A detailed description of the different types of simulations performed with Ichthyop are provided in section 2.2.1 below.

### 2.2 Simulations

Two main sets of simulations were performed to analyze the transport and accumulation patterns of floating litter in the Mediterranean. The first set used the offline coupling between MMF and Ichthyop as described above, being referred here as ‘*lagrangian simulation*’ (*LS*). The second set of simulations only used the MMF capabilities to simulate the dispersion of a floating inert tracer being introduced in the Mediterranean Sea with the freshwater flow from the rivers. This later simulation is called ‘*tracer simulation*’ (*TS*) through the rest of this text (see Table 1 for details).

**Table 1:** Full list of performed simulations with their main characteristics, full name, short acronym, total integration time, coastal behavior of the particles and initial time.

Full description	Acronym	Integration time	Coast behavior	Initialization time
Bouncing, long term simulation	Bo-LS	18 y	Bouncing	1^st^ January 2000
Beaching, long term simulation	Be-LS	18 y	Beaching	1^st^ January 2000
January, bouncing, short term simulation	J-Bo-SS	1 y	Bouncing	1^st^ January each year (2000–2017)
March, bouncing, short term simulation	M-Bo-SS	1 y	Bouncing	1^st^ March each year (2000–2017)
October, bouncing, short term simulation	O-Bo-SS	1 y	Bouncing	1^st^ October each year (2000–2017)
Tracers simulation	TS	18 y	–	1^st^ January 2000

#### 2.2.1 Lagrangian simulations (LS)

For *LS*, the starting point was a homogeneous release of 50000 particles throughout the entire Mediterranean basin. This initial number represents a mean initial density of 0.021 particle km^-2^ or ∼1.7 particles within each node of the model grid, limited by computational power. All particles are deployed at the surface and are simulated to have a density of 950 kg m^-3^, *i.e*., floating particles. This density is in the lower range of the ‘heavy polymers’ according to Pereiro et al. (2018) and it is close to the density of micro-plastics reported by Moret-Ferguson et al. (2010).

The direct effect of the wind drag on the individual particles is not considered, as small floating plastic fragments are considered to move under the sea surface in agreement with previous approaches modelling the movement of marine floating microplastic (*e.g*., Lebreton et al., 2012; Mansui et al., 2015; Iwasaki et al., 2017). Therefore, our simulations are addressed on microplastic-type pollution or to those large plastic items which remain mainly below the sea surface. Large, highly-buoyant items being mostly above the sea surface (*e.g.*, plastic bottles with air inside, polystyrene boxes) are, thus, not accounted for in the present approach. In general, and according to the analysis of Pereiro et al. (2018) and Yoon et al. (2010), the wind drag for particles of a density comparable to that in our simulations should be always smaller than 1%, so our approach would likely introduce very limited bias.

Two different *LS* were explored in relation to the particles' reaction to the coastline; one allowing them to ‘bounce’ back (*Bo-LS*) while in the other the particles are ‘beached’ (*Be-LS*). In addition, three sub-sets of simulations were carried out for the *Bo-LS*, releasing particles at three different times of the year. Hence, a series of eighteen 1-year simulations were done by seeding 50000 particles throughout the Mediterranean with different starting times in order to account for the possible effect of the initialization time. In the first set of simulations, particles were released every 1^st^ of January and the model was run until 31^st^ December of each given year, from 2000 to 2017 *(J-Bo-LS*). For the second set, particles were released on the 1^st^ May of year ‘*t*’ and model integration was done until 30^th^ of April of year ‘*t+1*’ (*M-Bo-LS*). The third set of simulations was run seeding particles on the 1^st^ of October of year ‘*t*’ and simulating their dispersion until 30^th^ September of year ‘*t+1*’ (*O-Bo-LS*). A ‘bouncing’ behavior was selected for the particles in all simulations, so no beaching was allowed.

#### 2.2.2 ‘Tracers’ simulation (TS)

The second type of simulation used the MMF to model the distribution of tracer input from river flow entering the Mediterranean (*TS*). The Framework for Aquatic Biogeochemical Models (FABM, Bruggeman and Bolding, 2014) was used to couple *on-line* a ‘tracer’ model to GETM, thus, simulating the advection of an inert tracer substance throughout the Mediterranean from 2000 to 2017. A constant concentration (=1 relative unit, r.u.) was imposed in all rivers. Initial concentration of the tracer in the Mediterranean Sea is set to zero and the tracer is simulated to be buoyant (floating velocity = 10 m d^-1^). As before, no direct wind drag is considered in the models.

## 3 Results

### 3.1 Surface currents dynamics

**Fig. 2:**
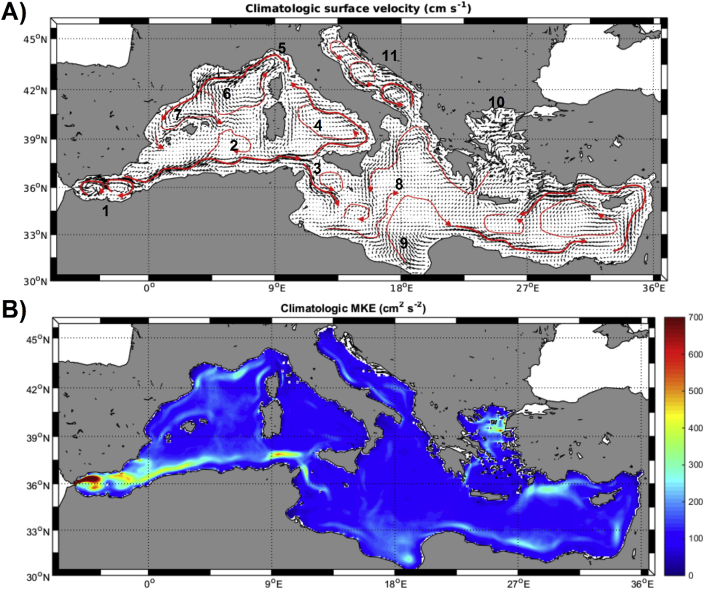
A) Climatological surface (0–10 m) velocity vectors (black arrows, cm s^-1^) with main surface currents highlighted with red arrows. B) Climatological surface (0–10 m) Mean Kinetic Energy (cm^2^ s^-2^). Numbers refer to the main sub-basins cited in the text: (1) Alboran Sea, (2) Ligurio-Provenzal basin, (3) Sicily Strait, (4) Tyrrhenian Sea, (5) Ligurian Sea, (6) Gulf of Lion, (7) Balearic Sea, (8) Ionian Sea, (9) Gulf of Sirte, (10) Aegean Sea, (11) Adriatic Sea. (For interpretation of the references to colour in this figure legend, the reader is referred to the Web version of this article.)

Our analysis firstly featured the surface velocity fields (averaged over the first 10 m of the water column) for the Mediterranean (Fig. 2a), which highlighted the main circulation patterns at basin scale (*e.g.,* Robinson et al., 1991; Pinardi et al., 2006; Poulain et al., 2012). The model closely fitted the main Mediterranean currents, proving its validity to drive floating plastic drift. Here, we describe the main circulation features outlined by our hydrodynamic model.

In the westernmost side (Alboran Sea), receiving the surface inputs of Atlantic water, there are two major anticyclonic gyres and the well-marked Almeria-Oran front. From there, the strong, coastal-attached Algerian Current develops eastwards with clear meandering, especially at the Ligurio-Provenzal basin. Reaching the Sicily Strait, the main current splits in two branches; one entering the Ionian Sea and the second flowing into the Tyrrhenian Sea. This later current follows the north-Sicilian coast veering northwards along the Italian coasts developing a general cyclonic circulation in the Tyrrhenian basin, entering then the Ligurian Sea at the north and creating the Northern Current that flows westwards along the French Riviera. Part of this current detaches from the coast after the Gulf of Lion region creating a large cyclonic cell in the north-western Mediterranean while its main vein continues along the shelf slope into the Balearic Sea. Upon reaching the Ibiza Strait, most of this current is deflected eastward along the northern coasts of the Balearic Archipelago with some residuals currents still moving southwards along the Spanish coasts.

The branch of the Algerian Current moving into the Ionian Sea develops in a series of connected cyclonic and anticyclonic gyres occupying mostly the Southern Mediterranean. A strong shelf-slope current continues from the Gulf of Sirte eastwards reaching the far-east coasts of the Mediterranean Sea where it turns northwards encircling Cyprus and returning westwards along the coasts of Turkey. Then, the main current moves along Crete's southern coast while two clear cyclonic gyres develop. Water flowing out of the Aegean Sea moves along the southern Greek coast into the northern part of the Ionian following the coast shape. Finally, a general cyclonic circulation is observed in the Adriatic Sea, with three well defined cyclonic cells.

The mean kinetic energy (MKE) map resulting from the surface circulation patterns described above shows where surface currents are stronger (Fig. 2b). Maximum levels of kinetic energy are simulated in the Alboran Sea where the Atlantic Jet enters the Mediterranean generating two anticyclonic gyres. The Almeria-Oran front and the Algerian Current are visible with high MKE values, as well as the Northern Current in the north-western part of the basin. At basin scale, MKE levels are lower in the Eastern than in the Western Mediterranean.

**Fig. 3:**
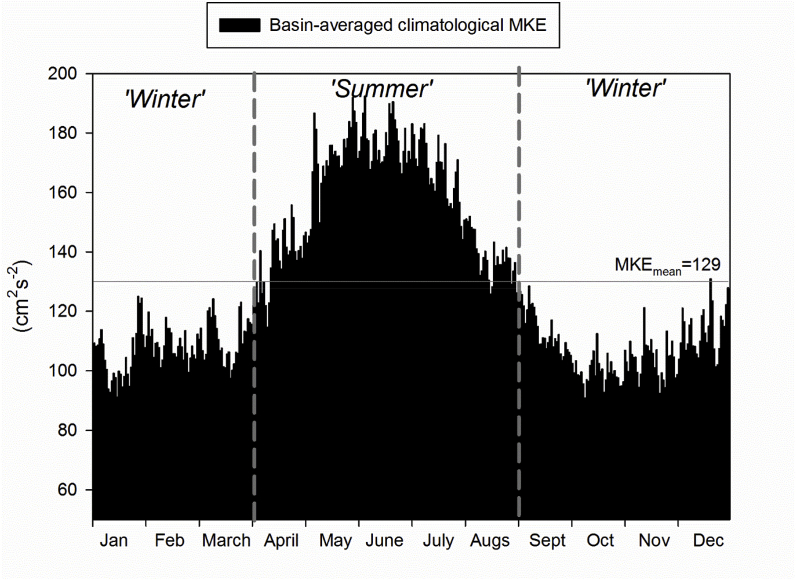
Basin-averaged monthly climatological MKE at surface (0–10 m). The total mean MKE is indicated with the horizontal gray lines. Vertical dashed bars indicate the different periods (‘extended summer’ and ‘extended winter’) used in the subsequent analysis (see text for details).

As climatologic circulation patterns simulated by the MMF for the surface of the Mediterranean Sea agree with previous knowledge (*e.g.,* Millot, 1999; Rio et al., 2007) we can use the long-time series of the simulation to analyze if there is some seasonality in the surface currents conditions. For this aim, we compute the Mean Kinetic Energy (MKE) over the entire basin by applying the following equation: MKE_i,j_ = ½*mean (*u*^2^_i,j_ + *v*^2^_i,j_), where *i,j* represents the position of each grid cell within the model domain and *u* and *v* the zonal and meridional surface current respectively. This computation is made for each time-step of the simulation and its average over the entire basin is calculated, creating a MKE time series. The mean annual cycle of this MKE series (Fig. 3) clearly shows maximum values around summer months and lower in autumn and winter. This seasonal pattern was divided in two periods, an ‘extended summer’ (from April to August) when MKE is above its annual mean value (horizontal line in Fig. 3) and an ‘extended winter’ below the mean (from September to March). A distinction into two rough ‘seasons’ has been used in previous studies (*e.g*., Zambianchi et al., 2017), although here, the threshold between seasons was set using a completely objective approach.

At spatial scale, winter MKE is only larger than the summer one in the western sector of Alboran Sea in coincidence with the winter acceleration of the Atlantic Jet (Fig. S1a, Macías et al., 2016). In summer, MKE levels are larger almost everywhere, especially along the main currents (Fig. S1c). It is also worth mentioning the appearance of a MKE maximum in the southern part of the Gulf of Sirte in summer.

### 3.2 Floating litter from open-sea sources (*LS*)

It has been already shown that, for the Mediterranean Sea, the length of model integration does make a difference in the results obtained for floating litter (Mansui et al., 2015; Zambianchi et al., 2017). Hereafter, we will present results of simulations done for two different time-horizons, multiyear simulations (integrated over 18 years) and annual simulations (done for each individual year within the period 2000 to 2017) as described in section 2.1.1 above.

#### 3.2.1 Long term (18 years) distributions (Bo-LS and Be-LS)

Two different 18-years simulations (from 2000 to 2017) described the movement of 50000 particles spread homogeneously throughout the Mediterranean Sea on the 1^st^ January 2000 (see details in section 2.2.1). The first simulation (*Bo-LS*) was done using the ‘bouncing’ behavior within Ichthyop, so all particles remain as free-floating during the entire integration period. In this type of simulation, when a particle is transported to a ‘dry’ cell at time *t+1* is ‘bounced’ back to its previous position, so it continues to be moved by the water currents until the simulation ends at December 2017.

**Fig. 4:**
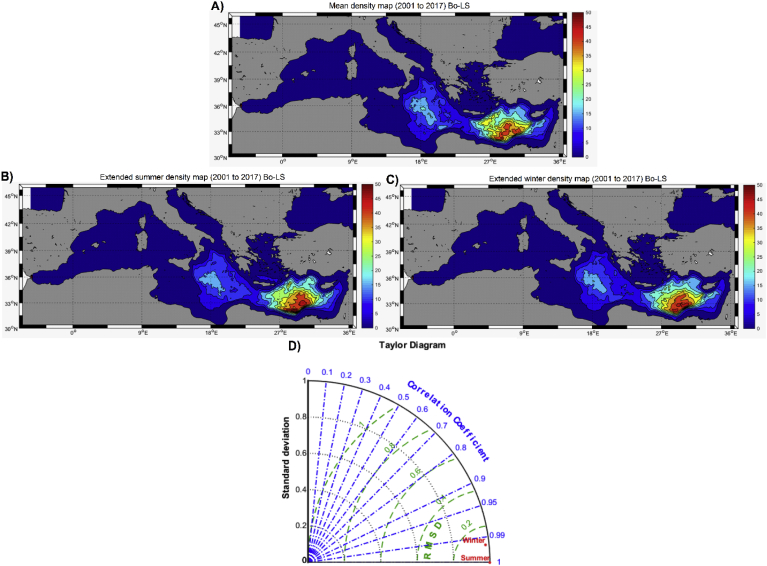
A) Mean climatologic particles distribution at surface for the last five years (2013–2017) in the ‘long term’ simulation with ‘bouncing’ behavior (Bo-LS, see details in the text) (# grid cell^-1^). B) Extended summer particles distribution (2013–2017) (# grid cell^-1^). C) Extended winter particles distribution (2013–2017) (# grid cell^-1^). D) Taylor Diagram of the comparison of maps in panels B) and C).

The averaged distribution of particles during the last five years of *Bo-LS* (*i.e*., 2013 to 2017) shows two accumulation zones, one located north of the Gulf of Sirte, in the mid-Ionian Sea, and a more dense accumulation zone to the south of Crete and Cyprus, in the Eastern Mediterranean (Fig. 4a). The maps for the extended summer and extended winter (Fig. 4b and c) shows similar distributions, with comparable accumulation zones. The similitude between seasons is further confirmed by the Taylor Diagram (Fig. 4d), where winter and summer (red points) are very close together, with a correlation coefficient over 0.9. There is a negative correlation between MKE and the final density of floating particles in this simulation (Fig. S2), with significant accumulations only happening in regions where MKE is below ∼200 cm^2^s^-2^.

**Fig. 5:**
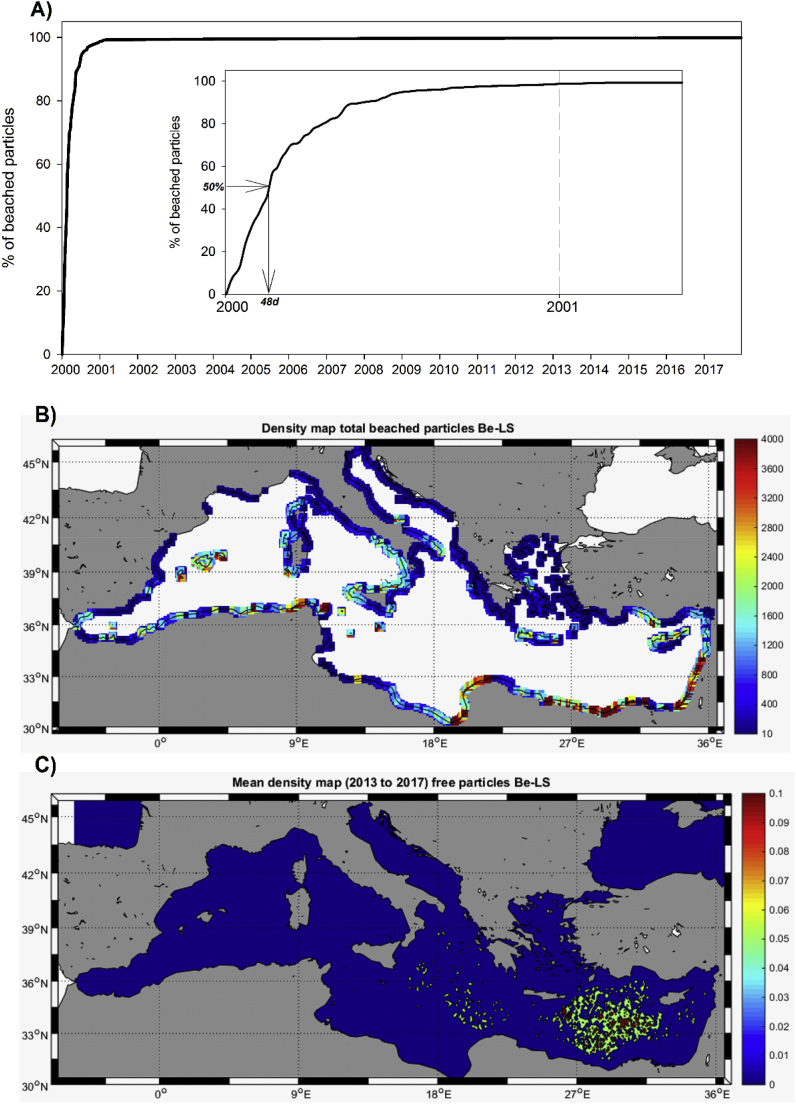
A) Percentage of stranded particles with time in the ‘long term’ simulation with ‘beaching’ behavior (Be-LS, see details in text). B) Density map of ‘beached’ particles (# km coast^-1^). C) Mean density of ‘free-floating’ particles for 2013–2017 period (# grid cell^-1^).

‘Beaching’ behavior was modeled in the second ‘long-term’ simulation (*Be-LS*). Therefore, if a particles reaches a non-water cell from time *t* to time *t+1* it is flagged as ‘*beached*’ and it remains in that dry position for the rest of the simulation. Numbers of beached particles derived from this particular simulation should be treated as the upper boundary of expected range since, in nature, a fraction of the items would be washed off back into the sea (*e.g.,* Liubartseva et al., 2018). Fig. 5a shows the percentage of initial particles reaching land for each time-step of the model run. After 1 year a very large percentage of the initial seeded particles (∼98.7%) have already been beached (inlet of this panel). For the remaining 17 years of simulation, only ∼1% more particles arrived to land (final percentage of beached particles ∼99.8%). Fig. 5b shows coastal regions receiving floating particles. The model predicts large concentrations on the southern coasts of the Eastern Mediterranean as well as in the easternmost coastlines. Within the Western Mediterranean, most affected coasts are those facing westwards (e.g., Corsica and Sardinia), the Algerian coast, Balearic Islands and western Italian façade. Relatively small amounts of floating litter reach the coasts of the north-western region from Liguria in Italy to the Balearic Sea in Spain.

Fig. 5c shows the final (stable) distribution of floating, free-particles in *Be-LS*. The spatial pattern is similar to those shown in Fig. 4, with accumulation zones in the central Ionian Sea and in the open-sea regions of eastern Mediterranean. The density of the accumulation zones is, however, much smaller than in the previous simulation, as only ∼0.2% of the initial particles remains on the sea surface (Fig. 5a).

### 3.3 Short term (yearly) distributions

Short term simulations (*J-Bo-SS, M-Bo-SS* and *O-Bo-SS*) inform about a scenario where the residence time of the particles on the sea surface is relatively short, within a temporal frame of 1 year. This 1 year integration time was chosen in order to explore persistent (>3 months) accumulation features reported by former works (Mansui et al., 2015) and to include the complete seasonal cycle.

**Fig. 6:**
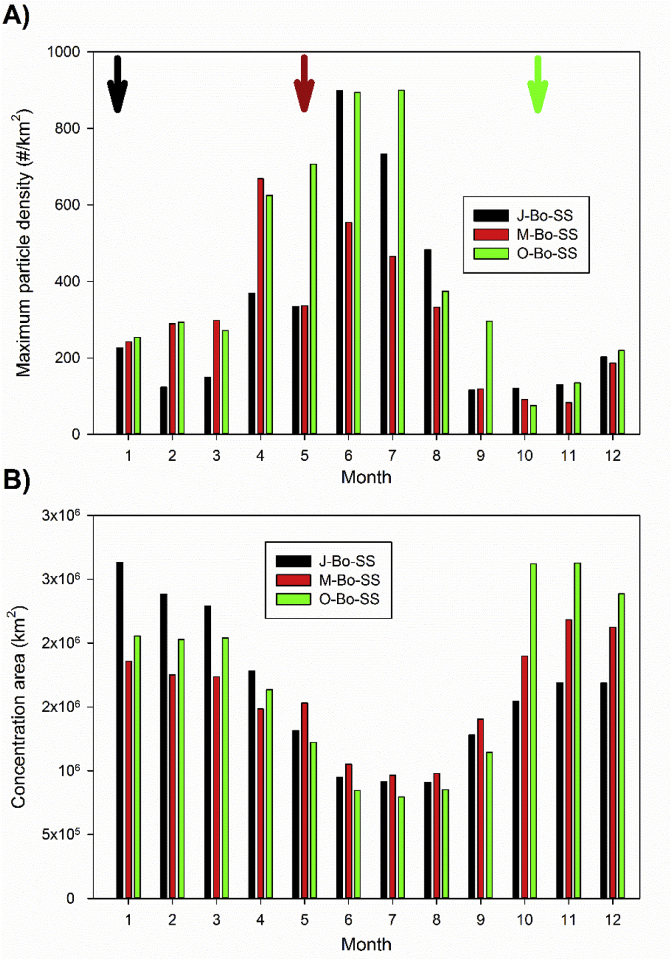
A) Maximum monthly climatogical concentration of particles for each of the ‘short-term’ simulations (see details in text). B) Mean climatological area where particles concentration is larger than the initial value for each of the ‘short term’ simulations. The vertical arrows in panel A) indicate the release times for the three sets of ‘short term’ simulations.

Monthly maximum particle concentrations (Fig. 6a) and the monthly area with concentration larger than initial (Fig. 6b) show a similar pattern for the three cases, with maximum concentrations being simulated during the ‘*extended-summer*’ months, and much lower concentrations during the rest of the year. Also, all simulations show that the area where there is effective concentration of particles (*i.e*., concentration larger than initial value) is smaller during the central months of the year. The consistency of the patterns regardless of the release date indicates that they are due to the internal dynamics of the currents/hydrological properties rather than the initial conditions of the simulations.

**Fig. 7:**
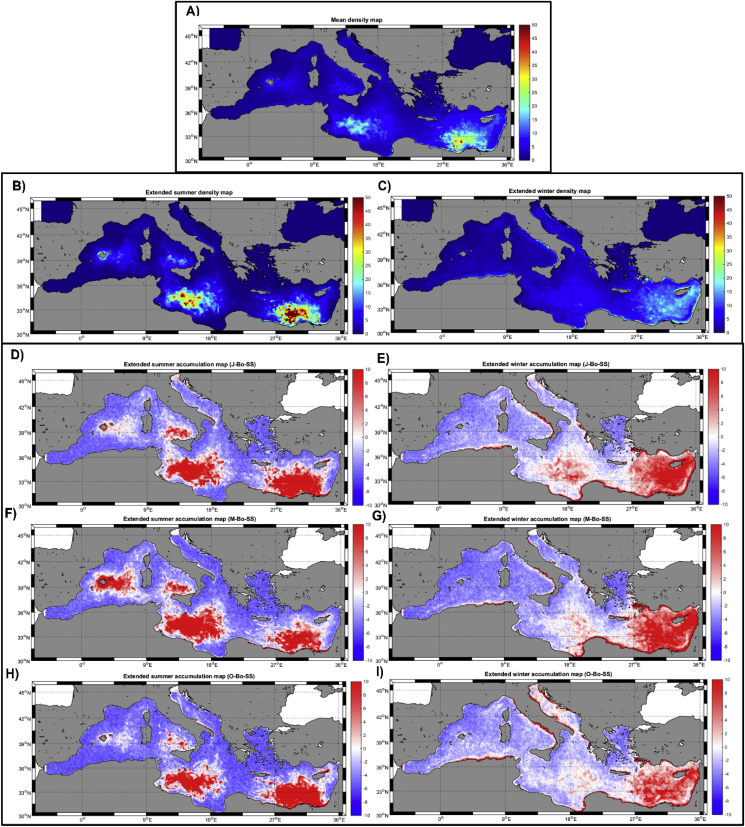
Climatological particle density maps (# grid cell^-1^) for all the ‘short term’ simulations for the whole year (A), for the ‘extended summer’ (B) and for the ‘extended winter’ (C) periods (see explanation in the text). Panels D) to I) climatological differences (# grid cell^-1^) with respect to the initial distribution for each season and for each individual ‘short-term’ simulation (see explanation in text).

**Fig. 8:**
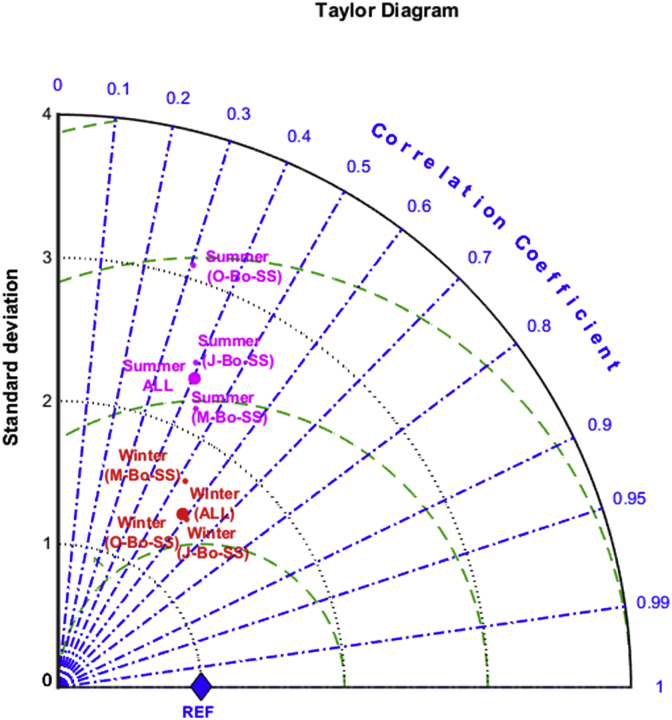
Taylor Diagram of the ‘extended summer’ (magenta symbols) and ‘extended winter’ (red symbols) for the different ‘short-term’ simulations (release of particles in January, May and October) and for their average distribution. (For interpretation of the references to colour in this figure legend, the reader is referred to the Web version of this article.)

The averaged climatological distribution of particles for all three set of simulations as well as for the ‘*extended summer’* and ‘*extended winter*’ seasons are shown in Fig. 7a–c. The time-averaged distribution (Fig. 7a) looks similar to that reproduced by the long-term simulation *Bo-LS* (Fig. 4a) but, in this case, differences on the spatial distribution for the two seasons are apparent. In the ‘*extended summer’*, floating particles tend to accumulate in four regions, the Liguro-Provenzal basin (around the Balearic Islands), the Tyrrhenian Sea, the south-western Ionian and the southern region of the Eastern Mediterranean (Fig. 7b). During the ‘*extended-winter’*, particles accumulate in the far eastern Mediterranean, with a smaller accumulation within the Gulf of Sirte (Fig. 7c). Both spatial distributions are statistically different as shown by the Taylor Diagram (Fig. 8). Moreover, these patterns are quite consistent through all simulations independently of the starting date (Fig. 7d–i). In all cases, the seasonal patterns are roughly repeated, again indicating that the choice of the starting date for the simulations is not significant. For all individual sets of simulations (*J-Bo-SS, M-Bo-SS* and *O-Bo-SS*), ‘summer’ and ‘winter’ distributions are different (red and magenta symbols in Fig. 8). Furthermore, seasonal differences are repeated within each individual year for each set of simulation (Fig. S3), demonstrating a strong consistency for the seasonal pattern that goes beyond the interannual variability in surface ocean conditions.

### 3.4 Floating litter from rivers (*TS* simulations)

Much of the marine floating litter comes with freshwater flow, either with wash-off water or via riverine discharges (Lebreton et al., 2012). This might be even more relevant for a basin such as the Mediterranean Sea, totally surrounded by land and receiving inputs from multitude of rivers (Liubartseva et al., 2018). Hereafter, we performed a last set of model runs simulating the dispersion of a floating tracer reaching the sea with the freshwater flow from the rivers.

**Fig. 9:**
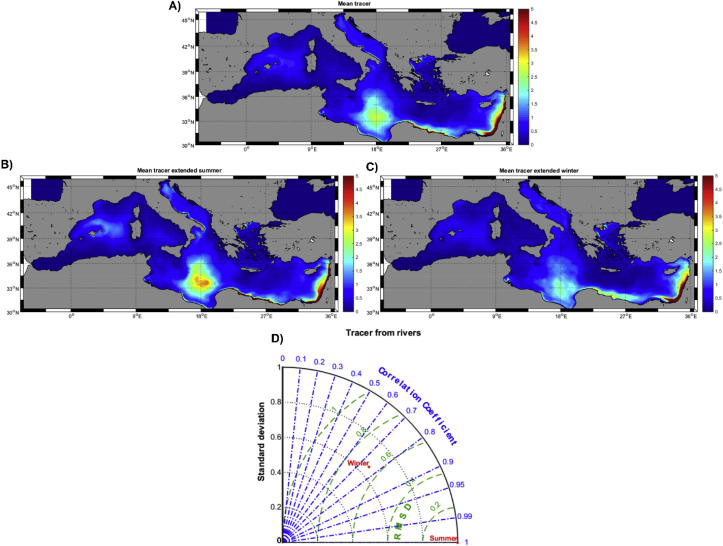
A) Mean climatologic tracer distribution at surface for the ‘tracers’ simulation (TS, see details in the text) (r.u. m^-3^). B) Extended summer tracer distribution (r.u. m^-3^). C) Extended winter tracer distribution (r.u. m^-3^). D) Taylor Diagram of the comparison of maps in panels B) and C).

Averaged concentration (in relative units m^-3^) of river-input tracer on the surface of the Mediterranean Sea shows higher accumulation in the southern Ionian Sea and especially towards the nearshore waters of the Southeastern Mediterranean (Fig. 9a). Comparing with the short term lagrangian distributions (*J-Bo-SS, M-Bo-SS* and *O-Bo-SS*), Ionian accumulation might resemble that one of the accumulations found in summer, while the high density of tracers in the southeastern nearshore waters could be related to the winter transport of floating particles toward the Eastern Mediterranean (Fig. 7). The Taylor Diagram shows statistically significant differences between ‘*extended summer*’ and ‘*extended winter*’ distributions (Fig. 9d). These seasonal differences were, however, minor with a reinforcement of the accumulation zone in the southern Ionian Sea in summer (Fig. 9b and c).

## 4 Discussion

Different numerical modeling simulations revealed, for the first time, a marked seasonal pattern of variability in the distribution of floating litter in the Mediterranean, arising from a consistent seasonality in MKE (Fig. 3). The existence of a seasonal cycle in surface current conditions has been already reported for several regions of the Mediterranean Sea, such as the Alboran Sea (Bormans et al., 1986; Macías et al., 2016), the Northern Current (Millot, 1999; Font et al., 1995; Macías et al., 2018a) or even for the overall Western Mediterranean (Pascual et al., 2014).

This annual cycle of surface currents in some Mediterranean regions has been related previously with the seasonality in the interchange through the straits (Millot, 1991) or linked to the manifestation of the seasonal cycle of the steric signal (Pascual et al., 2014). There have been previous quantitative assessments of the kinetic energy seasonality for the Mediterranean basin. The model of Roussenov et al. (1995) showed peaks of kinetic energy integrated throughout the whole water column in March and August, while the model of Fernandez et al. (2005) found opposite results, with maximum values in winter and minimum in summer. The divergence in the reported seasonal cycles could be related to the the way the kinetic energy computation is done (i.e., integration depth) or due to uncertainties associated with the modelling tools, which limit the comparison of results. We must note that we analyze MKE up to 10-m depth in order to address the surface layer containing most of marine floating particles (Kooi et al., 2016) while previous works reported integrated MKE values. Nonetheless, even in the model by Fernandez et al. (2005) where MKE seasonal cycle seems to follow the opposite pattern as the one described here, they also showed that seasonality of water transport through concrete sections and straits, in most cases, attained maximum values in summer.

From the analysis of the stratification conditions (squared buoyancy frequency, N^2^) in the upper 50 m of the water over 18 years (Fig. S4a), we found that, as expected, N^2^ values are larger during the central months of the year, indicating a much higher vertical stratification. At the same time, averaged wind intensity over the Mediterranean Sea (Fig. S4b) shows that, in summer, winds could be as strong as in the middle of the winter. These two figures indicate that in the central months of the year the wind forcing on the surface ocean is quite strong and that it acts on a fairly thin layer due to the high vertical stratification. This combination of factors could help explaining the seasonal cycle of surface current intensity shown in Fig. 3 and discussed in several other previous works (Bormans et al., 1986; Millot, 1999; Pascual et al., 2014).

Given the wide seasonal differences found in kinetic energy of the Mediterranean Sea, it is reasonable to assume that the transport and accumulation patterns of floating litter in the basin show different seasonal patterns. However, the results of the long-term simulations (*Bo-LS*) do not show such differences (Fig. 4b and c), with similar accumulation zones simulated for both seasons. Indeed, the high density regions in Fig. 4b and c match with those reported from previous modelling works (*e.g.,* Lebreton et al., 2012) which have shown accumulation regions in the central Ionian Sea and in the southern part of the Eastern Mediterranean. In other cases, however, models have simulated slightly different accumulation regions in the Mediterranean using land-based inputs of litter (Liubartseva et al., 2018). High concentration zones in Liubartseva et al. (2018) were located closer to main rivers (as input sources) and in the central Ionian Sea (as in our case). We hypothesize that the former accumulation zones are here missed because no riverine inputs are used in the *LT* simulations.

The beaching pattern arising from our simulations (Fig. 5b) fits with results previously reported by Mansui et al. (2015), with large amounts of beached litter typically found in the eastern-basin coasts (Golik and Gertner, 1992). Beaching distribution also shows that the westward facing coasts of islands are typically more polluted than eastward facing ones (*e.g*., Corsica, Sardinia and Sicily) although in some other archipelagos the southern (in Balearic) or northern (Crete and Cyprus) façades are more affected. Litter pollution in beaches and coasts has economic impacts derived from aesthetic issues affecting tourism (Jang et al., 2014), habitat degradation (Carson et al., 2011) or reducing the provisioning of ecosystems services (Liquete et al., 2016). Predictions from simulation tools as the MMF could help to guide field surveys and contribute to planning and managing potential mitigation programs.

On the one hand, our set of simulations has clearly confirmed that integration time of the model does impact the final results, as already shown by Mansui et al. (2015) with 1-year integration simulation showing different results to long-term (18-year) results. On the other hand, it is remarkable that the mean monthly distributions of floating litter are minimally dependent on the time of release, whether it happen in winter, spring or fall (Fig. 6, Fig. 7), for the annual simulations. Several hot spots of floating litter are predicted to form across the Mediterranean in ‘summer’, while accumulation zones are less apparent in winter in all model runs. Such consistency demonstrates that the observed patterns are not an artifact of the initial conditions but rather a feature emerging from the seasonal hydrodynamics of the Mediterranean basin. Moreover, we found a clear year-on-year consistency (Fig. S3). It is noticeable that, in all short term lagrangian simulations and in the tracer simulation, the Balearic Archipelago appears as an accumulation zone during the summer months. Touristic activity is the key industry in this region, so the presence of floating litter (and other floating particles such as jellyfish) in their coasts during high-occupancy months could strongly impact local and regional economy.

In winter, floating litter is mainly accumulated towards the Eastern Mediterranean, although this accumulation is more spread and less dense than the hot spots formed in the summer season. The addition of “litter” into the Mediterranean through the river network (*TS*, Fig. 9) mainly reinforce the winter distribution pattern, stressing the accumulation of litter in the south-eastern nearshore waters. Interestingly, the highest concentrations of floating plastics measured in the Mediterranean Sea has been reported along the Israeli nearshore waters, in close agreement with our simulations (van der Hal et al., 2017).

To date, published models on particle tracking for the Mediterranean lack of consistent validation, mostly due to the limited coverage in space and time of the oceanographic surveys. Seasonal comparisons of floating plastic debris in the Mediterranean has been only addressed on the Western basin (Suaria and Aliani, 2014; Campana et al., 2018; Arcangeli et al., 2018), where our model predicts more and denser aggregations in summer months (Fig. 7). Campana et al. (2018) sampled the NW Mediterranean along 3 years (2013–2016) and found significantly higher anthropogenic litter concentration during spring-summer than in fall-winter. They also identified the highest concentrations in the Balearic Sea, coincident with our model predictions (Fig. 7). This high concentration region in the vicinity of the Balearic Archipelago was also mentioned by Ruiz-Orejon et al. (2018) who, indeed, hypothesized that this accumulation area was created by the predominant hydrodynamic features on the surface layer, presenting higher litter density during summer months than in winter (Ruiz-Orejon et al., 2019). An additional seasonal analysis was reported by Arcangeli et al. (2018), who described a clear seasonality with higher concentration of debris in summer for all sampled areas except for the Adriatic. This is also in concordance with our simulations (Fig. 7) as no significant differences among seasons is expected for the Adriatic Sea. Finally, Suaria and Aliani (2014) found considerably higher average density in June than in October for year 2013. Hence, although further data are required for model calibration and validation, available data converge on the observation of denser litter aggregations during the summer months, which closely match with our predictions.

This seasonal variability in retention structures could in part explain the differences between models and observations reported for this regional sea (Cozar et al., 2015; Ruiz-Orejon et al., 2016; Suaria et al., 2016). Higher sampling coverage accounting for possible seasonal basin-scale variability in surface transport seems to be required to validate the existence of accumulation zones of floating litter. Likewise, further improvements in the modelling capabilities are necessary in order to obtain a more accurate description of the floating plastic litter distribution.

It is worth commenting that the particle tracking model used in the present contribution is an exploratory approximation to the behavior of plastics. We are not including key processes such as the breaking-down of the pieces or changes in buoyancy (for example due to bio-fouling). It has been, indeed, proposed that sinking and bottom accumulation could be one of the main processes involved in the elimination of floating plastic from the surface of the ocean (Cozar et al., 2004; Kaiser et al., 2017). There are, also, some missing surface processes such as tidally-induced currents and Stokes drift that have been shown to modify significantly the result of particle tracking models in specific oceanic regions (e.g., Zhang, 2017; Iwasaki et al., 2017; Onink et al., 2019). Hence, further refinements of the model internal dynamics and more realistic data-based inputs forcing of litter should be developed in the near future.

In spite of these relevant shortcomings, the MMF tools used here have contributed to advance the understanding of the linkage between the hydrodynamics and the distribution of floating litter in the Mediterranean Sea. In this sense, the MMF has already been applied to create scenarios of probable changes on the hydrodynamic and biogeochemical conditions of the Mediterranean Sea within the context of climate change (e.g., Macías et al., 2015; 2018a; 2018b). The coupling of these scenarios on hydrodynamic changes with the particle tracking modules could help to understand if potential accumulation regions/periods would likely change in the future. These type of future scenario simulations together with different management options (e.g., related to waste management or plastic use) could provide valuable information to stakeholders aiming to the preservation of the Good Environmental Status (GES) of European waters as sought by the Marine Strategy Framework Directive (MSFD, 2008/56/EC) of the European Commission.
