# Supplementary material for: Surface water circulation develops seasonally changing patterns of floating litter accumulation in the Mediterranean Sea. A modelling approach
Source: Mar Pollut Bull. 2019 Dec;149:110619. doi: 10.1016/j.marpolbul.2019.110619 (PMC6891234; doi:10.1016/j.marpolbul.2019.110619)
Supplement: Multimedia component 1 [file mmc1.docx]

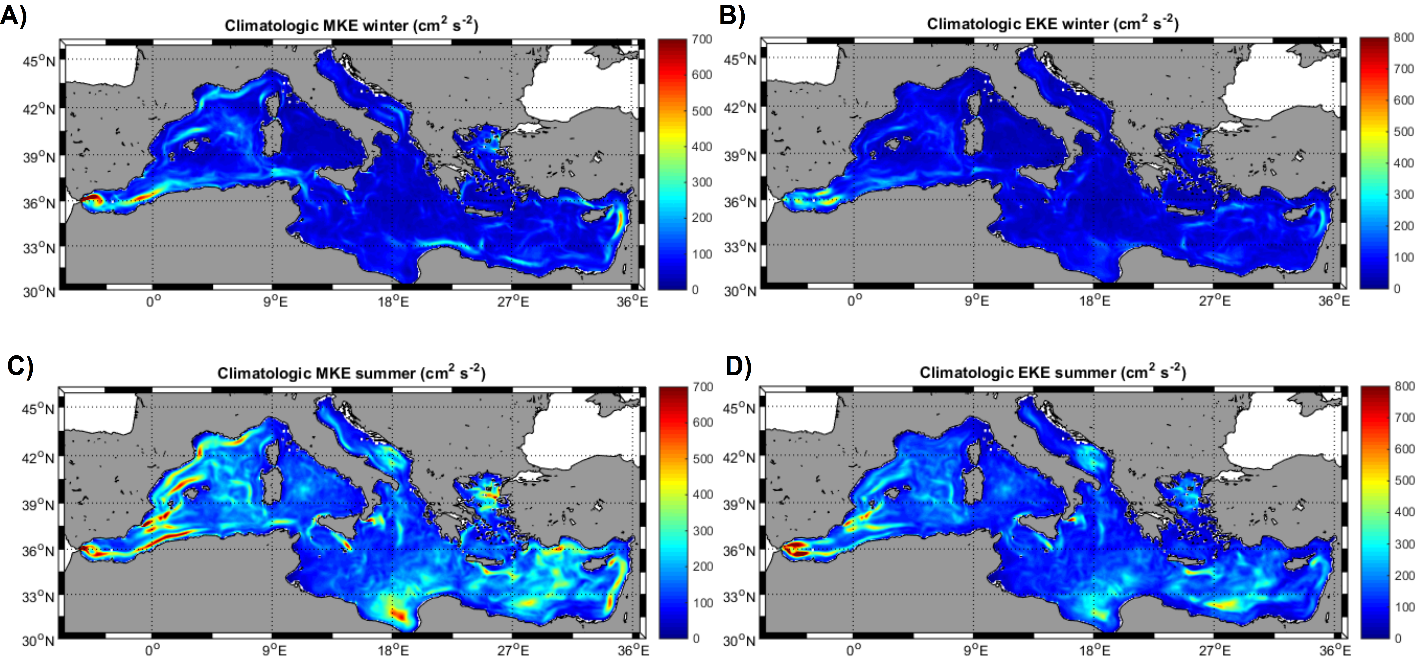


Figure S1. MKE and EKE distributions for the ‘extended summer’ and ‘extended winter’ seasons.





Figure S2. Scatter plot of MKE versus particle density for the continuous run simulation with no-beaching behavior (*Bo-LS*).


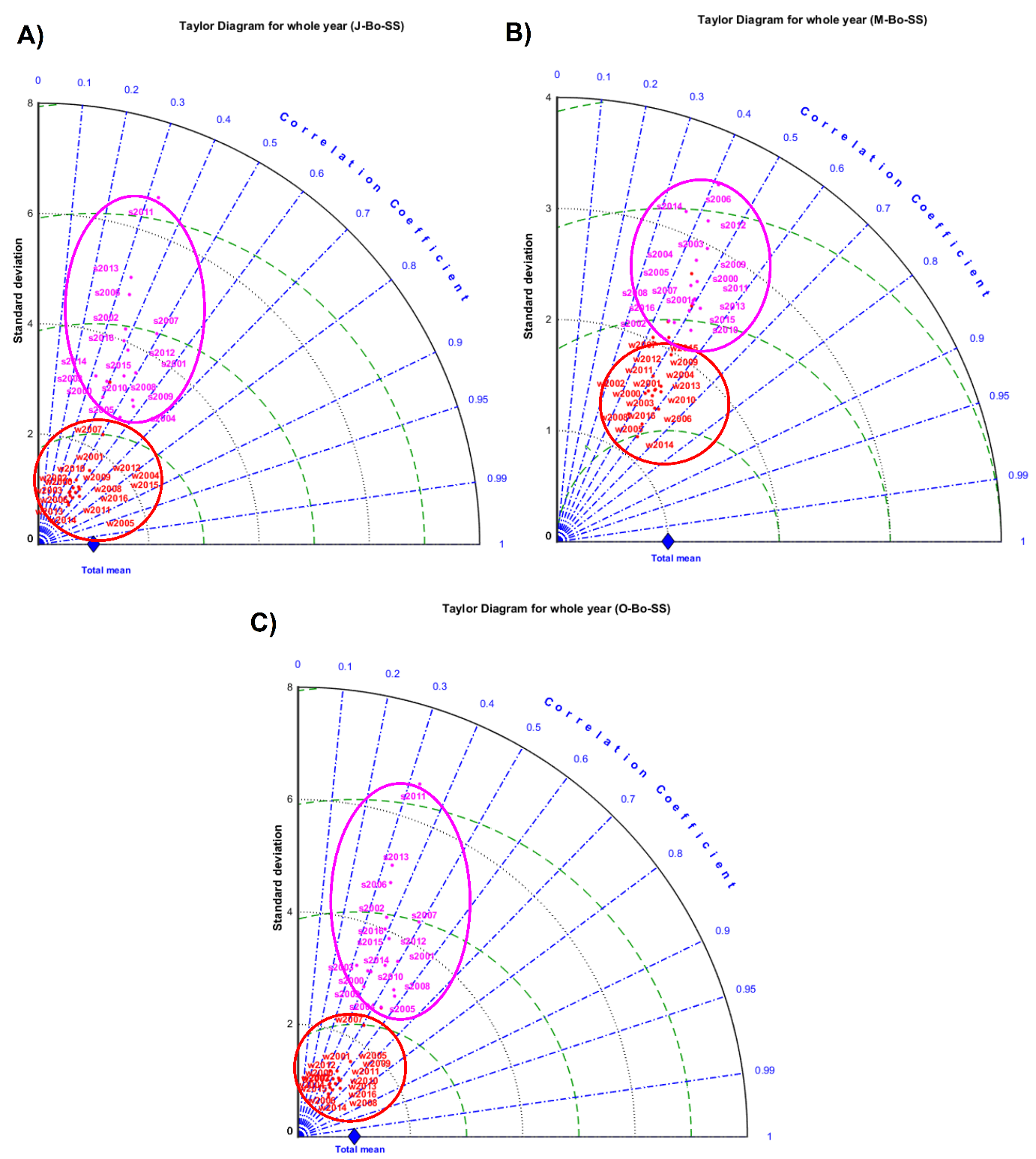


Figure S3. Taylor Diagrams for the different ‘yearly’ simulation showing the ‘summer’ (magenta symbols) and ‘winter’ (red symbols) distributions for each of the 18 individual years.





Figure S4. A) Mean monthly stratification index for the entire Mediterranean basin. B) Mean monthly wind intensity over the entire Mediterranean Sea
